# Adhesion molecules in subjects with COPD and healthy non-smokers: a cross sectional parallel group study

**DOI:** 10.1186/1465-9921-14-47

**Published:** 2013-05-01

**Authors:** Kristin Blidberg, Lena Palmberg, Anna James, Bo Billing, Elisabeth Henriksson, Ann-Sofie Lantz, Kjell Larsson, Barbro Dahlén

**Affiliations:** 1 Lung and Allergy Research, National Institute of Environmental Medicine, Karolinska Institutet, Box 287, Stockholm, SE-171 77, Sweden; 2 Experimental Asthma and Allergy research, Division of Physiology, The National Institute of Environmental Medicine, Karolinska Institutet, Stockholm, Sweden; 3 Division of Respiratory Medicine and Allergy, Dept of Medicine, Karolinska University Hospital Huddinge, Stockholm, Sweden

**Keywords:** Adhesion molecules, Chronic Obstructive Pulmonary Disease, Neutrophils, Sputum, Bronchoialveolar lavage fluid

## Abstract

**Background:**

The aim of the study was to investigate how the expression of adhesion molecules changes as neutrophils migrate from the circulation to the lung and if these changes differ between non-smoking subjects and smokers with and without COPD.

**Methods:**

Non-smoking healthy subjects (n=22), smokers without (n=21) and with COPD (n=18) were included. Neutrophils from peripheral blood, sputum and bronchial biopsies were analysed for cell surface expression of adhesion molecules (CD11b, CD62L, CD162). Serum, sputum supernatant and BAL-fluid were analysed for soluble adhesion molecules (ICAM-1, -3, E-selectin, P-selectin, VCAM-1, PECAM-1).

**Results:**

Expression of CD11b was increased on circulating neutrophils from smokers with COPD. It was also increased on sputum neutrophils in both smokers groups, but not in non-smokers, as compared to circulating neutrophils.

Serum ICAM-1 was higher in the COPD group compared to the other two groups (p<0.05) and PECAM-1 was lower in smokers without COPD than in non-smoking controls and the COPD group (p<0.05). In BAL-fluid ICAM-1 was lower in the COPD group than in the other groups (p<0.05).

**Conclusions:**

Thus, our data strongly support the involvement of a systemic component in COPD and demonstrate that in smokers neutrophils are activated to a greater extent at the point of transition from the circulation into the lungs than in non-smokers.

## Introduction

Chronic obstructive pulmonary disease (COPD) is characterised by a chronic airway inflammation, tissue destruction and irreversible airflow obstruction. Neutrophils are one of the main effector cells in COPD and there are numerous studies that show increased neutrophil numbers in bronchoalveolar lavage (BAL) fluid, sputum, in bronchial biopsies and blood [1-4]. Thus, the inflammatory process in COPD is not only localised to the lungs and COPD is today generally recognised as a systemic disease [5].

Neutrophils are one of the first cells to arrive at the site of infection where they exhibit a broad functional repertoire in counteracting harmful agents. The migration of the neutrophils from the circulation to the site of infection or injury is a carefully regulated series of events involving adhesion molecules, chemoattractants and cytokines. The migration has been described as a multistep process including slow rolling, adhesion strengthening, intraluminal crawling and finally paracellular, or transcellular, migration through the endothelium [6]. The initial rolling is mediated by L-selectin (CD62L) expressed on neutrophils, and E-selectin and P-selectin expressed on the endothelium. The main ligand for these selectins is P-selectin glycoprotein ligand (PSGL)-1 (CD162) expressed both on neutrophils and certain endothelial cells. Next, firm adhesion is mediated through, for example, macrophage antigen-1 (Mac-1/CD11b) expressed on neutrophils and its ligand intercellular adhesion molecule (ICAM)-1 expressed on the endothelium [7]. The third step, intravascular crawling involves CD11b and other β2-integrins [8]. Prior to the final step, transendothelial migration, vascular cell adhesion molecules (VCAM)-1 and ICAM-1 form so called docking structures on the endothelial cells [7,8]. For the final transendothelial migration, interaction between two platelet/endothelial cell adhesion molecule (PECAM)-1 expressed at the borders of the endothelial cells and on neutrophils is essential [9,10].

In addition to its role as an adhesion molecule, CD11b is used as a marker of neutrophil activation as its activation is linked to several neutrophil functions such as the oxidative burst, phagocytosis and release of proteolytic enzymes [11]. Studies showing increased neutrophil expression of Mac-1 in subjects with COPD have led to the suggestion that neutrophils in COPD are activated to a greater degree compared to neutrophils in healthy subjects [12,13]. Our knowledge of adhesion molecule function mainly stems from studies of vascular inflammation and animal models, and the understanding of their role and significance in COPD is limited.

In the current study, we examined the expression of soluble adhesion molecules on neutrophils from different compartments (blood, sputum, bronchoialveolar (BAL) fluid and bronchial biopsies). Taken together, the patterns observed in the different locations may provide a better understanding of the alterations that occur in the neutrophils during transition from the circulation to the airway. Moreover, a further aim was to investigate whether these neutrophil characteristics differ between smokers with COPD, smokers without COPD and healthy non-smokers.

## Materials and methods

### Subjects and study design

Twenty-two healthy non-smokers, 21 current smokers without chronic airflow limitation and 18 current smokers with COPD were recruited by advertisement in daily press. Smokers were included in the COPD group if post-bronchodilator FEV_1_ /FVC was < 0.7 and FEV_1_ was >40% of the predicted normal value. Smokers were allocated to the non-COPD group if they had a post-bronchodilator FEV_1_ /FVC of >0.70. Healthy never-smokers with normal spirometry made up the control group. A history of asthma or other pulmonary or allergic disease constituted exclusion criteria (Table 1). The patients were clinically stable at the time of the study and were not included if they had experienced an airway infection during the past 14 days prior to the study. In the COPD group 7 subjects inhaled tiotropium and 6 subjects inhaled a combination of steroids and long-acting beta-agonists on a regular basis. All medication was withheld 48 hours prior to the investigations.

**Table 1:** Subjects’ characteristics

	**Controls**	**Smokers without COPD**	**Smokers with COPD**
**No.**	22	21	18
**Age (years)**	55.0	54.0	61.9
	51.6-58.4	50.6-57.4	58.6-65.1
**Gender (Female/Male)**	7/15	11/10	13/5
**Smoking (pack-years)**	-	32.7	38.1
		26.3-39.1	33.5-42.7
**FEV**_**1 **_**%predicted post-bronchodilatation**	112.1	103.0	66.2
	106.8-117.5	97.4-108.6	59.2-73.3
**FEV**_**1**_**/FVC post-bronchodilatation**	0.79	0.76	0.56
	0.77 -0.82	0.73-0.79	0.52-0.59
**DLCO %predicted**	91.8	73.2	48.2
	84.9-98.7	67.3-79.1	40.3-56.1

Data are presented as mean and 95% confidence intervals.

Blood samples were collected from all subjects and 12 subjects from each group underwent bronchoscopy, including bronchoalveolar lavage followed by at least three biopsies, and sputum induction on two separate days. There was a minimum of 10 days between bronchoscopy and sputum induction.

The study was approved by the local ethics committee at Karolinska Institutet, Stockholm, Sweden (D-nr 2005/733-31/1-4) and all subjects provided their written informed consent.

### Bronchoalveolar lavage (BAL) and biopsies

Bronchoscopy was performed as previously described [14]. After pre-medication with scopolamine, analgesics and local anaesthesia with xylocaine the bronchoscope was wedged in a middle lobe segmental bronchus and 5 aliquots of 50 mL isotonic saline were instilled into the airway tree and gently sucked back. The 5 aliquots of lavage fluid were pooled and then centrifuged. The supernatant was divided into aliquots and kept at -70°C until analysis. Slides were also prepared by cyto-centrifugation and stained using May-Grünwald Giemsa reagent. Bronchial mucosal biopsies were taken from the subcarinas of an upper lobe segment.

### Sputum induction and processing

Sputum induction was performed as previously described [15]. The cell-pellet was re-suspended in 2 mL PBS and kept on ice until antibody staining for flow cytometric analysis. Slides were also prepared by cyto-centrifugation and stained using May-Grünwald Giemsa reagent. For differential cell counts 300 non-squamous cells were assessed.

### Blood

Peripheral blood was drawn in ethylene diamine-tetra-acetic acid (EDTA) vacutainer tubes (BD Biosciences, San Jose, CA, USA) for flow cytometric assessment of cell surface markers and differential cell counts and in vacutainer tubes without anticoagulant (BD Biosciences) for serum analysis of soluble adhesion molecules. For differential cell counts MultiTEST CD45PerCP/CD3FITC/CD4APC/CD8PE (BD Bioscience) and TruCOUNT^TM^ tubes (BD Biosciences) were used according to the manufacturer’s instructions. Absolute counts of lymphocytes, monocytes, neutrophils, eosinophils and basophils were determined by analysis in MultiSET (BD Biosciences).

### Adhesion molecules on peripheral blood and sputum neutrophils

Whole blood and sputum cells were stained with titrated amounts of anti-CD11b PE, anti-CD62L PE or anti-CD162 PE together with anti-CD45 PerCp. Isotype matched anti-bodies were used as negative controls. All antibodies were from BD Biosciences. Samples were analysed using a FACS Calibur^TM^ (BD Biosciences) flow cytometer with CellQUEST^TM^ software. Results are presented as mean fluorescence intensity (MFI=monoclonal antibody - matched isotype control).

### Soluble adhesion molecules

Soluble adhesion molecules were analysed in subjects where representative samples from all three compartments (serum, sputum supernatant, BAL fluid) were obtained (n=12).

Soluble adhesion molecules were analysed in serum, sputum supernatants and BAL-fluid (only ICAM-1) using the Adhesion 6-plex FlowCytomix^TM^ Multiplex kit (Bender Medsystems, Vienna, Austria). Analyses were performed according to the manufacturer’s instructions using FACSCalibur^TM^ flow cytometer (BD Biosciences) and FlowCytomix Pro2.2 Software (Bender Medsystems, Vienna, Austria). Detection ranges were as follows: sE-selectin: 4–3,000 ng/mL, sICAM-1: 5-4,000 ng/mL, sICAM-3: 11–8,000 ng/mL, sPECAM-1: 4–3,000 ng/mL, sP-selectin: 19–14,000 ng/mL and sVCAM-1 5-4,000 ng/mL.

The following adhesion molecules were analysed in BAL fluid by Duo Set ELISA (RD Systems Europe, Abingdon, UK ) according to the manufacturer’s instructions. The standard range was sE-selectin: 93–6,000 pg/mL, sICAM-3: 31–2,000 pg/mL, sP-selectin: 125–8,000 pg/mL and sVCAM-1 31-2,000 pg/mL. Soluble PECAM-1 was analysed using ELISA (Abnova, Taipei City, Taiwan) according to the manufacturer’s instructions. The standard range was 156–10,000 pg/mL.

### Processing and staining of bronchial biopsies

Biopsy specimens were embedded in glycol methacrylate (GMA) and processed as previously described [16]. Sequential biopsy sections (2 μm) were cut from the GMA blocks with a Leica RM 2165 microtome (Leica Microsystems, Germany) and floated onto 0.2% ammonia solution prior to adherence to glass microscope slides (Superfrost®, Thermo Scientific).

Biopsies were stained using the Envision kit (DAKO, Sweden) according to the manufacturer’s instructions with minor modifications. Briefly, incubation times for the antibodies were increased to 1 hour and the double stain block was extended to 5 minutes. The following antibody dilutions were used: anti-neutrophil elastase (NE) 1:300 (DAKO), anti-CD11b 1:200 (Abcam, Cambridge, UK), anti-CD62L 1:100 (BioVision Research Products, Mountain View, USA) and anti-CD162 1:200 (Epitomics, Burlingame, USA).

### Statistics

Lung function data are presented as mean and 95% confidence intervals. Data on cell distribution and adhesion molecules are presented as median and interquartile range. Comparisons between groups were made using the Kruskal-Wallis test followed by a Mann–Whitney test when appropriate and by Spearman Rank Order. The Wilcoxon Signed Rank Test was used for within group comparisons between compartments, these comparisons were made only in subjects with samples from both of the relevant compartments.. A p-value below 0.05 was considered significant. For non-parametric statistical calculations samples below the detection limit in the Adhesion 6-plex FlowCytomix™ Multiplex kit were assigned a value of 2 ng/mL. Data was analysed using STATISTICA 9 (StatSoft, Inc., Sweden).

## Results

### Cell distribution in blood, sputum and BAL

The number of circulating neutrophils was significantly higher in the COPD group compared to the group of smokers without COPD (p=0.04) and healthy controls (p=0.01, Table 2). The cell numbers and the cell distribution in sputum did not differ between the groups (Table 2).

**Table 2:** Cell distribution in blood, bronchoalveolar lavage and induced sputum

		**Controls**	**Smokers without COPD**	**Controls vs Smokers without COPD p-value**	**Smokers with COPD**	**Controls vs Smokers with COPD p-value**
**Blood (cells x10**^**3**^**/mL)**	**Monocytes**	512	597	ns	616	ns
		363-741	482-674		516-790	
	**Neutrophils**	2914	3633	0.2	4072	**0.01**
		2121-4188	2942-3889		3456-5392	*0.04
	**Lymphocytes**	1828	2442	ns	2189	ns
		1675-2752	1857-3148		1942-2624	
	**Eosinophils**	184	255	ns	269	ns
		117-249	135-318		236-334	
**BAL (cells x10**^**6 **^**/L)**	**Cells x10**^**6**^**/L**	114	290	**0.0001**	281	**0.002**
		83.2-129	259-523		156-351	
	**Macrophages**	91.3	264	**0.0001**	242	**0.001**
		79.1-115.7	225-498		143-305	
	**Neutrophils**	3.8	12.4	**0.03**	8.4	**0.04**
		0.4-5.1	3.5-21.5		3.6-21.7	
	**Lymphocytes**	4.7	13.3	**0.003**	10.2	0.2
		3.6-8.9	11.2-23.5		3.3-19.8	
	**Eosinophils**	0	0	0.2	2.4	**0.01**
		0-0.2	0-1.1		0.3-4.5	
**Sputum (cells/mg)**	**Cells/mg**	715	603	ns	652	ns
		514-908	284-866		409-1370	
	**Macrophages**	250	218	ns	234	ns
		155-603	53.5-444		136-424	
	**Neutrophils**	396	415	ns	501	ns
		157-490	132-591		343-786	
	**Lymphocytes**	23.2	9.3	ns	14.9	ns
		18.2-31.8	7.2-11.3		4.5-55.6	
	**Eosinophils**	0	1.3	ns	8.2	ns
		0-5.0	0-2.6		0.6-16.8	

Data are presented as median and 25^th^ – 75^th^ percentile. Comparisons between groups were made using Kruskal-Wallis test followed by Mann–Whitney test when appropriate. * indicate p-value for comparison between smokers with and without COPD.

In BAL fluid, the numbers of macrophages and neutrophils were higher in the two smoker groups compared to the non-smoking control group (p<0.05). Furthermore, eosinophils were increased in the COPD group compared to the non-smokers (p=0.01) and lymphocytes were increased in the group of smokers without COPD as compared to the healthy non-smokers (p=0.003, Table 2).

### Adhesion molecules on blood and sputum neutrophils

**Figure 1:**
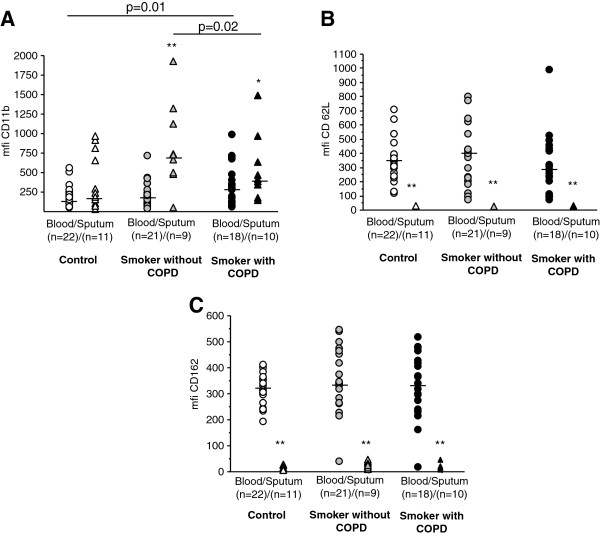
**Surface expression of adhesion molecules on blood and sputum neutrophils.** Surface expression of **A**) CD11b, **B**) CD62L and **C**) CD162 on blood neutrophils and sputum neutrophils measured by flow cytometry. Results are presented as mean florescence intensity (mfi). P-values indicate comparisons between groups within the same compartment. Cell numbers were not sufficient for flow cytometric analysis in all sputum samples, the analysed numbers are indicated in the figure.

Expression of CD11b was increased on blood neutrophils from COPD subjects compared to non-smoking controls (p=0.01, Figure 1A). Moreover, CD11b expression on sputum neutrophils was higher in smokers without COPD compared to the COPD group (p=0.02) and to non-smoking controls (p=0.05). There were no significant differences in CD62L or CD162 expression between the groups (Figure 1B, C).

Expression of CD11b was higher on sputum neutrophils compared to peripheral blood neutrophils from smokers with (p=0.009) and without COPD (p=0.01). There was no difference in CD11b expression between the two compartments in the healthy controls. In contrast, expression of CD62L and CD162 was significantly lower on sputum neutrophils compared to peripheral blood neutrophils in all groups (p<0.05).

**Figure 2:**
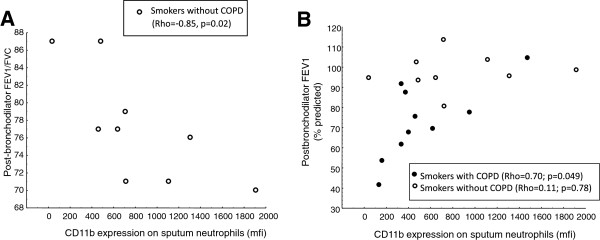
**Correlations between lung function and CD11b expression on sputum neutrophils. A**: Relation between FEV_1_/FVC and CD11b expression in smokers without chronic airflow limitation. **B**: Relation between disease severity, assessed by FEV_1_ in % of predicted value, and CD11b expression.

There was a negative correlation between expression of CD11b on sputum neutrophils and FEV_1_/FVC in smokers without COPD (Rho=−0.85, p=0.02) and a correlation between expression of CD11b on sputum neutrophils and FEV_1_ (% predicted) in the COPD group (Figure 2).

### Soluble adhesion molecules

**Figure 3:**
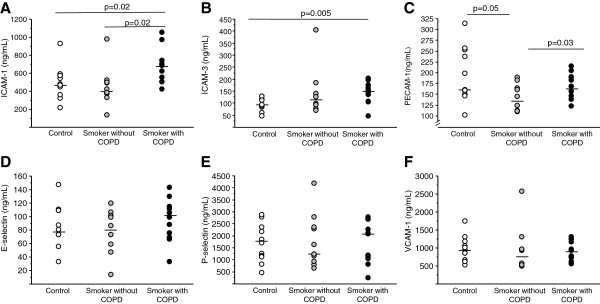
**Soluble adhesion molecules in serum.** Levels of soluble adhesion molecules **A**) ICAM-1, **B**) ICAM-3, **C**) PECAM-1, **D**) VCAM-1, **E**) E-selectin and **F**) P-selectin measured in serum from controls (n=12), smokers without COPD (n=12) and smokers with COPD (n=12). Soluble adhesion molecules were measured in subjects where samples from all compartments (blood, sputum and BAL) were available. Results are presented as ng/mL. P-values indicate comparisons between groups.

*In serum* the concentration of sICAM-1 was higher in the COPD group than in the other two groups (p=0.02) and sICAM-3 was higher in the COPD group than in healthy non-smokers (p=0.005). Furthermore, sPECAM-1 was lower in the smokers without COPD than in the smokers with COPD (p=0.03). There were no other significant differences between the groups with regard to serum levels of soluble adhesion proteins (Figure 3).

*In BAL-fluid* sICAM-1 was lower in the COPD group than in the other groups (p<0.05), also PECAM-1 was higher in smokers without COPD compared to the other two groups (p<0.05). Otherwise soluble adhesion molecules in BAL fluid did not differ between the groups (Table 3). Soluble E-selectin and P-selectin were only detectable in three and four BAL fluid samples respectively (data not presented).

**Table 3:** Soluble adhesion molecules in BAL fluid and sputum

		**Controls**	**Smokers without COPD**	**Smokers with COPD**
**BAL fluid**	ICAM-1	94.8	94.3	47.6*^, §^
	(ng/mL)	84.3-122.7	78.0-149.9	29.1-86.1
	ICAM-3	15.1	16.1	12.4
	(pg/mL)	9.6-26.2	8.7-26.1	10.6-18.8
	PECAM-1	285.5	758.6^§,^	351.4*
	(pg/mL)	194.2-684.1	525.7-1037.1	217.1-512.9
	VCAM-1	281.5	122.4	114.4
	(pg/mL)	118.5-484.9	103.9-204.7	75.2-293.9
**Sputum supernatant (ng/mL)**	ICAM-1	16.6	16.6	33.6
		<5.0-42.7	<5.0-34.4	<5.0-51.9
	ICAM-3	17.1	20.3	28.7
		14.1-60.5	<11.0-43.2	10.3-49.3
	E-selectin	< 4.0	< 4.0	12.5
		<4.0-17.7	<4.0-13.0	<4.0-23.1
	P-selectin	< 19.0	< 19.0	< 19.0
		<19.0 - 40.9		< 19.0 -25.5
	PECAM-1	12.2	9.0	13.4
		6.6-23.8	< 4.0-13.8	< 4.0-18.0
	VCAM-1	7.1	< 5.0	8.5
		5.1-7.8	<5.0-8.7	<5.0-12.0

Results are expressed as ng/mL; median and 25^th^- 75^th^ percentiles.

§ p< 0.05 compared to non-smoking controls.

* p< 0.05 compared to smokers without COPD.

*In sputum supernatants* the levels of soluble adhesion molecules did not differ between the groups (Table 3).

### Bronchial biopsies

**Figure 4:**
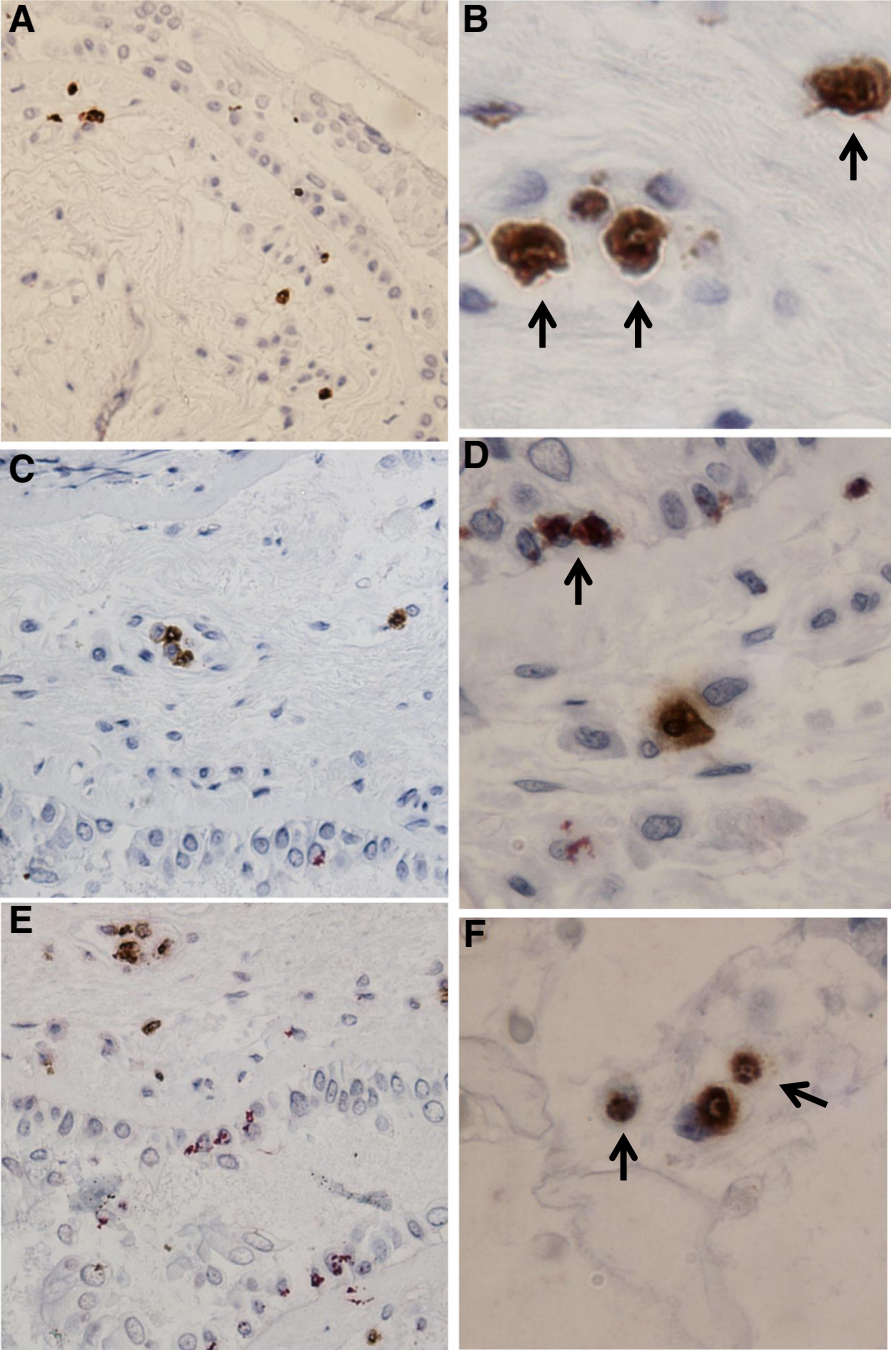
**Expression of adhesion molecules in bronchial biopsies.** Co-localisation of neutrophil elastase and **A,B**) CD11b, **C,D**) CD62L and **E,F**) CD162 in bronchial biopsies from a patient with COPD. Neutrophil elastase positive cells are stained in brown; adhesion molecule positive cells are stained in red. Arrows indicate cells double stained cells positive for neutrophil elastase and the respective adhesion molecule. Sections are counterstained with haematoxylin. Original magnification for figure **A, C** and **E** is x200 and original magnification for **B,E** and **F** is x500.

Bronchial biopsies from three subjects in each group were double-stained and showed the presence of neutrophils expressing CD11b, CD62L and CD162. Representative images are displayed in Figure 4.

## Discussion

In the present study it was shown that CD11b expression on neutrophils is enhanced in association with their transition from the blood to the airways in smokers. This upregulation was observed irrespective of the presence of chronic airflow limitation, but was not shown in non-smokers. Furthermore, CD11b expression on circulating neutrophils was enhanced in patients with COPD compared to non-smokers. This finding demonstrates that even circulating neutrophils are activated in COPD and that smoking is associated with a further activation of airway neutrophils that is not observed in non-smokers. In addition it was shown that, while CD11b expression is enhanced on airway neutrophils from smokers, the expression of the adhesion proteins CD62L and CD162 on neutrophils almost vanishes when entering the airways of both smokers and non-smokers. We also showed that the levels of soluble ICAM-1 and ICAM-3 in serum are increased in COPD, confirming systemic inflammatory activity in this disease [17].

Although an increased CD11b expression on sputum neutrophils has previously been shown in COPD [13] this is, to our knowledge, the first time that CD11b expression in neutrophils has been studied in different compartments within the same COPD patient. The two groups of smokers are fairly well matched with regard to cumulative tobacco exposure and there is a substantial difference in lung function between the smokers with and without COPD, indicating that comparisons between the smoker groups may be related to the presence of airway obstruction. It is, however obvious that even smokers who did not display chronic airflow limitation and thus had not developed COPD according to current diagnosis criteria, were not healthy as diffusion capacity was clearly impaired in this group compared with healthy non-smokers. This finding indicates that pathological processes in the lungs, as a consequence of smoking, may develop along different lines in different individuals.

Our data showed an increased CD11b expression on sputum neutrophils from smokers without COPD and that CD11b expression on sputum neutrophils exceeded that of blood neutrophils in smokers, but not in non-smokers. This likely indicates that neutrophils from smokers, irrespective of airflow obstruction, become activated in connection with the transition from the circulation to the airways, a phenomenon that does not occur in non-smokers. In addition, sputum neutrophils from smokers without COPD are more activated than neutrophils from smokers with COPD. Smoke exposure of isolated neutrophils *in vitro* activates neutrophils as it causes increased expression of β_2_-integrin (CD18) and decreased CD62L expression [18]. One may speculate that the activation of sputum neutrophils caused by smoking may be attenuated due to exhaustion of the immune system in subjects with COPD, a speculation that is supported by the finding of a correlation between CD11b expression on sputum neutrophils and disease severity, as assessed by FEV_1_ (% of predicted). In line with this, expression of CD11b is down-regulated during apoptosis and while the apoptosis rate of circulating neutrophils has been shown to be unaltered in COPD, an increased apoptosis rate has indeed been reported in sputum neutrophils from subjects with COPD [19,20]. Further studies on neutrophil functions in this context have to be performed. Interestingly, FEV_1_/VC-ratio has previously been shown to be inversely correlated with CD11b expression on sputum neutrophils in subjects who were current or ex-smokers, irrespective of significant airflow obstruction [13]. Our findings are in agreement with these data in smokers without COPD, but are not confirmed in the smokers with COPD. This may lend further support to the notion that sputum neutrophils from subjects with COPD might have an impaired function compared to neutrophils from smokers without COPD.

Neutrophil expression of CD11b does not only attract attention as an adhesion molecule but it is also considered a marker of neutrophil activation. In the current study we showed that CD11b expression is increased on circulating neutrophils from subjects with COPD as compared to healthy controls. This is in agreement with earlier studies [12,21] and emphasises the systemic component of the inflammation in COPD as we demonstrated that even circulating neutrophils from patients with COPD displayed signs of activation. Whereas expression of CD11b increase on activated neutrophils, CD62L is shed upon activation [22]. Although there was no difference in CD62L surface expression between the groups in the current study, CD62L was significantly lower on neutrophils from sputum as compared to neutrophils from blood. Thus the CD62L data support the CD11b data, and suggest that airway neutrophils are further activated in relation to circulating neutrophils.

While neutrophil numbers in BAL fluid and sputum are often increased in COPD, bronchial biopsies from subjects with COPD show varying results with regard to neutrophil numbers [1-3,23,24]. It has been hypothesised that this discrepancy between different compartments is due to the rapid migration of neutrophils through the tissue in order to reach their primary destination in the airway lumen. In addition, increased neutrophil numbers have been suggested to be more characteristic of the inflammation present in the small airways [25,26]. In the current study neutrophil numbers in BAL fluid were increased and there was a similar trend in sputum.

Neutrophils expressing CD11b have previously been shown to be increased in the submucosa of subjects with COPD compared to control smokers [27]. In this study, the presence of CD11b staining was confirmed and immunohistochemical staining of bronchial biopsies showed neutrophils expressing also CD62L and CD162.

In the current study, neutrophil expression of CD162 did not differ between the groups, but there was a lower expression on sputum neutrophils compared to blood neutrophils. Contradictory to this finding, a small study of subjects with varying stages of COPD (GOLD I-IV) found increased CD162 expression on circulating neutrophils in the COPD group [28]. The ligand of CD162, P-selectin, has received attention as a marker of platelet activation and systemic inflammation. In one study it was found that platelets were activated in COPD, but this was not reflected by differences in soluble P-selectin [29]. This is in line with the current results where no differences in serum P-selectin were detected between the groups. However, other studies support a role for P-selectin as a marker of systemic inflammation in COPD and P-selectin has been associated with impaired lung function in a large cross-sectional study [30]. Also, Ferroni et al. found increased plasma levels of P-selectin in subjects with COPD as well as an inverse relation between P-selectin and PaO_2_[31]. It is possible that a larger sample size is needed to demonstrate a difference between groups although this would also limit its usefulness as a marker of systemic inflammation.

E-selectin is only expressed on activated endothelium and together with soluble ICAM-1 soluble E-selectin is sometimes considered a sign of endothelial activation. The percentage of E-selectin positive vessels in bronchial biopsies as well as serum E-selectin has been shown to be increased in COPD [32,33]. However, the current study found no significant difference in serum levels and it was below the detection limit in both sputum and BAL fluid samples.

Soluble ICAM-1 and sICAM-3 in serum were increased in smokers with COPD as compared to healthy non-smoking subjects. This finding is in agreement with those of Riise et al. [32] who found increased levels of circulating ICAM-1 in COPD patients with FEV_1_ between 60 and 70% of predicted value, i.e. similar to the patients in this study. Walter et al. also found increased levels of soluble ICAM-1 in serum from patients with moderate COPD, FEV_1_ 60 – 70% of predicted value [30]. Walter et al. also found a relationship between circulating ICAM-1 levels and disease severity, as assessed by FEV_1_[30], but this association was not demonstrated by Riise et al. [32]. In contrast to those findings Noguera et al. showed lower serum levels of ICAM-1 in COPD than in healthy non-smokers [21]. In that study patients with severe COPD, with FEV_1_ on average 33% of predicted value, were studied. The main sources of ICAM-1 are bronchial epithelial cells and endothelial cells [33,34]. Endothelial cells respond to pro-inflammatory stimuli by an increased production of sICAM-1 [34,35]. Elevated levels of TNF and other pro-inflammatory cytokines is a common feature of COPD which likely is of importance for the increased sICAM-1 levels observed in COPD. It could thus be hypothesised that there is an association between disease severity and the levels of ICAM-1 in mild and moderate COPD and that severe COPD, characterised by loss of functional pulmonary capillaries and airway mucosal cells, is associated with decreased production and release of adhesion molecules into the circulation.

Previously, Riise et al. showed an increase of sICAM-1 in bronchial lavage fluid [32]. In agreement with this observation, a trend towards higher sICAM-1 levels was observed in the sputum supernatants from the COPD group. Considering the 50-fold difference in volume between our BAL technique and their small volume bronchial lavage it is reasonable to relate the small volume bronchial lavage to sputum supernatants as they both represent the central airways. The role of sICAM-1 in the lung is not fully understood but both human studies and animal models indicate that alveolar epithelial cells are an important source of the sICAM-1 found in BAL fluid [36]. Moreover, it has been shown that sICAM-1 can induce neutrophil-mediated cytotoxicity [37] and activate lung macrophages [38]. It is therefore possible that the decreased sICAM-1 levels in BAL fluid, observed in the COPD group, could be related to an increased binding of sICAM-1 to different effector cells. In addition, it appears that ICAM-1 is differentially regulated in the bronchial epithelial cells and alveolar epithelial cells, with a more marked increase of ICAM-1 in response to stimuli observed in bronchial epithelial cells [36,39].

Expression of PECAM-1 on the endothelium has been shown to be of importance for transendothelial migration of neutrophils as the blocking of PECAM-1 abolishes transendothelial migration [40]. Soluble PECAM-1 can bind endothelial PECAM-1 and thus prevent neutrophils from transmigrating [40]. However, other adhesion molecules have been shown to be involved in transmigration and the adhesion molecules involved may change depending on stimulus and disease [41]. In the current study a tendency towards lower serum sPECAM-1 in smokers was found irrespective of airflow obstruction, with a significantly lower level in smokers without COPD as compared to the COPD group. It could be speculated that the lower levels in the smoker group are caused by sPECAM-1 binding to endothelial PECAM-1 as part of a protective mechanism, a mechanism that has failed in the COPD group. In BAL fluid sPECAM-1 was higher in smokers without COPD as compared to both other groups.

The available literature on the role of soluble adhesion molecules in COPD is contradictory. Most studies have investigated serum levels; although some authors have studied BAL fluid. There is large variation in severity of disease between the studies and there is also a difference in the techniques used to analyse the samples which makes direct comparisons difficult.

In conclusion, our data show that neutrophils, from COPD patients, retain, and further enhance, the state of activation that is observed in circulation, even after migration into the lungs. While the general role of soluble adhesion molecules in COPD still requires further investigations, some molecules, such as serum ICAM-1, appear to be reliable markers of the systemic inflammation in COPD.

## Competing interests

None of the authors have any conflicts of interest to declare.

## Authors’ contributions

KB performed flow cytometry work, collected and analysed data, drafted the manuscript. ASL coordinated the study, handled all contact with research subjects and managed sample collection. AJ, EH, BB performed bronchoscopies, performed biopsy work and helped drafting the manuscript. KL, BD, LP conceived of the study, participated in its design and coordination and were involved in drafting the manuscript. All authors read and approved the final manuscript.
